# Estimated Costs of Severe Adverse Drug Reactions Resulting in Hospitalization in the Veterans Health Administration

**DOI:** 10.1001/jamanetworkopen.2021.47909

**Published:** 2022-02-10

**Authors:** Sherrie L. Aspinall, Michelle Vu, Von Moore, Rong Jiang, Anthony Au, Mark Bounthavong, Peter A. Glassman

**Affiliations:** 1 VA Center for Medication Safety, Pharmacy Benefits Management Services, Hines, Illinois; 2 Health Economics and Outcomes Research, Optum Life Sciences, Eden Prairie, Minnesota; 3 VA Health Economics Resource Center, Palo Alto, California; 4 VA Pharmacy Benefits Management Services, Washington, DC

## Abstract

This quality improvement study uses Veterans Health Administration (VHA) data to estimate the costs of severe adverse drug reactions resulting in or contributing to hospitalizations.

## Introduction

Prior studies on adverse drug reactions (ADRs) have focused on severe events requiring hospitalization^1,2,3,4^; many of these studies were small, involving few medical centers.^2,3^ Information about costs by medication, symptom, or drug-symptom pair (eg, lisinopril-angioedema) is limited. Within the Veterans Health Administration (VHA), VHA Directive 1070 requires reporting of ADRs to the Veterans Affairs (VA) Adverse Drug Event Reporting System (ADERS),^5^ and these events can be integrated with other VHA clinical and economic data to inform decisions that may facilitate the prioritization of interventions to mitigate harm. We sought to estimate total medical costs for spontaneously reported severe ADRs by drug-symptom pair that resulted in or contributed to hospitalizations.

## Methods

This retrospective quality improvement study was performed from the VHA perspective. Outpatient-onset ADRs reported as severe in VA ADERS from fiscal years 2014 to 2018 were included (Figure). Reports contained details regarding patient, drug(s), event date, ADR symptoms coded using Medical Dictionary for Regulatory Activities terms at the Preferred Term (MedDRA PT) level, and setting.^5^ The Edward Hines Jr VA Hospital institutional review board approved the study and waived the requirement for informed consent because non–human participant data were used. This study followed the Standards for Quality Improvement Reporting Excellence (SQUIRE) reporting guideline.

**Figure.**
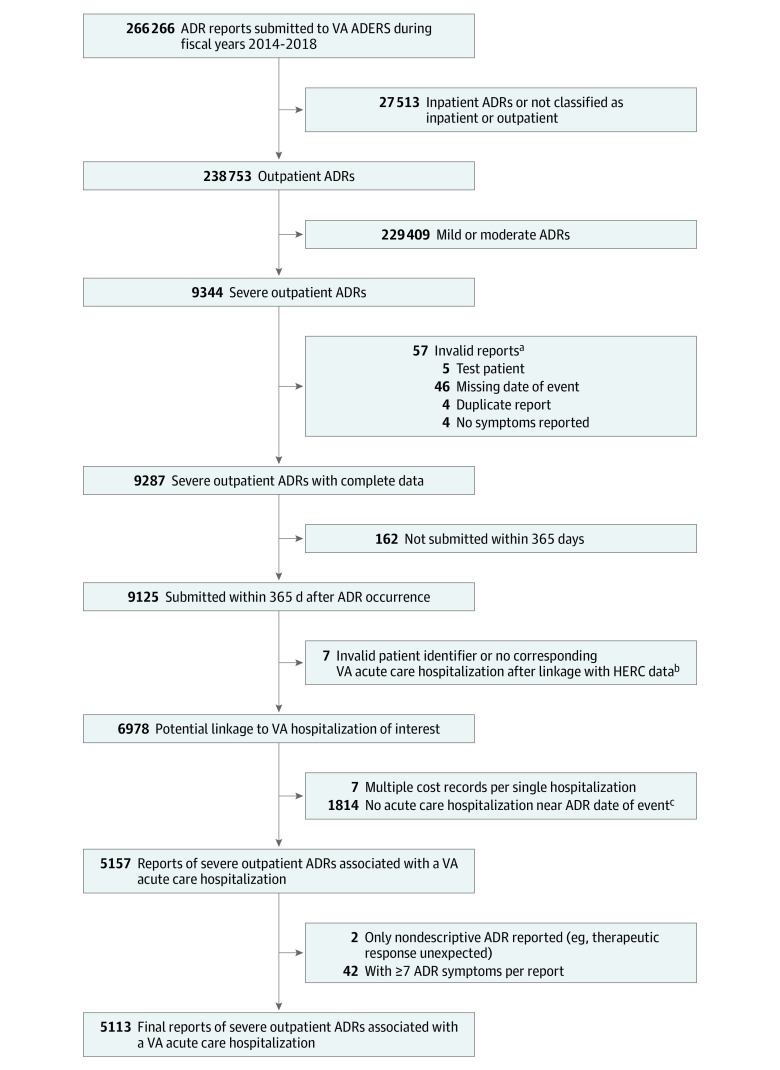
Identification of Severe Outpatient Adverse Drug Reactions (ADRs) Associated With Hospitalization Within the Veterans Health Administration ADERS indicates Adverse Drug Event Reporting System; HERC, Health Economic Resource Center; VA, Veterans Affairs. ^a^One patient was excluded because of missing date, test patient, and no ADR symptoms reported. ^b^No corresponding VA acute care hospitalization was reported during fiscal years 2013 through 2018 (the report may have been entered in VA ADERS within 365 days after admission). ^c^Matched with hospitalization based on proximity with the date of the ADR. The ADR date, which may have been estimated by the reporter, was within 14 days of a hospital stay or during a hospital stay.

Included ADRs from VA ADERS were linked to the likely corresponding VHA hospitalization and total costs based on patient identifiers and event date proximity (Figure). The VA Health Economic Resource Center Average Cost data sets provided hospitalization cost estimates using methods for predicting Medicare reimbursement.

In the VA ADERS reports, each medication was paired with all MedDRA PTs. Costs of ADRs by drug-MedDRA PT (ie, symptom) pair were summarized using mean, median, and measures of variance (SD and IQR). Costs were adjusted to 2018 US dollars using the Consumer Price Index. Analyses were performed using SAS, version 9.4 (SAS Institute Inc). Details are given in the eMethods in the Supplement.

## Results

The study included 5113 outpatient ADR reports from 4880 veterans. The mean (SD) patient age was 67.7 (12.2) years; most patients were male (4628 [94.6%]) and White (2622 [53.7%]). A total of 2792 of the 5113 outpatient ADR reports (54.6%) that resulted in or contributed to VHA hospitalizations included 1 symptom, and 3918 (76.6%) involved 1 medication.

The Table shows hospitalization costs for drug-symptom pairs with 25 or more reports (36 of 4026 unique pairs [0.9%]). The most reported drug-symptom pairs were lisinopril-angioedema (406 reports [7.9%]), warfarin-hemorrhage (311 [6.1%]), and warfarin–gastrointestinal hemorrhage (246 [4.8%]). Hydrochlorothiazide/lisinopril-angioedema had the lowest median cost during fiscal years 2014 through 2018 at $6951 (IQR, $4720-$10 510). Enoxaparin-hemorrhage had the highest median cost at $29 535 (IQR, $21 231-$44 236).

**Table.** Cost of Hospitalization for Drug-MedDRA PT Pairs With 25 or More Reports From FY 2014 Through FY 2018^a^

Drug-MedDRA PT pair	Reports, No. (patients, No.)	Costs summarized for FYs 2014-2018, $		Costs for FY 2018, $	
		Median (IQR)	Mean (SD)	Median (IQR)	Mean (SD)
Total	5113 (4880)	NA	NA	NA	NA
Lisinopril-angioedema	406 (404)	8431 (5318-12 650)	12 138 (13 060)	11 181 (8854-16 457)	16 107 (14 684)
Warfarin-hemorrhage	311 (304)	18 736 (11 207-35 454)	29 614 (30 242)	20 918 (13 370-37 056)	27 933 (22 845)
Warfarin-gastrointestinal hemorrhage	246 (242)	16 449 (11 269-26 163)	22 388 (20 233)	19 526 (14 784-29 532)	23 642 (13 426)
Warfarin–international normalized ratio increased	151 (148)	19 281 (10 086-38 184)	31 291 (33 034)	21 902 (14 410-64 487)	37 743 (33 603)
Warfarin-anemia	123 (120)	15 810 (10 449-25 481)	22 478 (21 330)	15315 (11207-27876)	21 237 (13 919)
Aspirin–gastrointestinal hemorrhage	81 (79)	13 818 (9106-24 192)	21 874 (24 424)	17 866 (15 944-19 727)	17 859 (3045)
HCTZ/lisinopril-angioedema	66 (66)	6951 (4720-10 510)	11 301 (12 355)	9045 (8436-10 362)	9362 (1665)
Apixaban–gastrointestinal hemorrhage	64 (64)	16 499 (9981-25 723)	21 145 (16 582)	17 397 (10 716-27 918)	22 292 (16 580)
Rivaroxaban–gastrointestinal hemorrhage	60 (60)	13 566 (10 997-20 761)	18 448 (12 887)	17 039 (13 405-25 104)	22 584 (17 577)
Ibuprofen–gastrointestinal hemorrhage	56 (56)	12 350 (9168-20 097)	17 148 (13 692)	13 402 (9973-23 944)	19 076 (12 778)
Lisinopril-hyperkalaemia	52 (52)	8462 (5457-15 255)	15 098 (22 391)	9053 (6265-10 699)	17 273 (23 470)
Naproxen–gastrointestinal hemorrhage	48 (48)	10 686 (7885-19 731)	17 359 (24 832)	11613 (9532-29 667)	17 238 (10 322)
SMX/TMP-hyperkalaemia	45 (45)	8827 (6574-15 185)	17 997 (33 086)	13 427 (7962-18 923)	14 328 (8349)
Aspirin-anemia	42 (41)	12 719 (8598-25 845)	18 901 (15 517)	22 442 (8724-36 159)	22 442 (19 399)
Dabigatran–gastrointestinal hemorrhage	39 (39)	12 492 (10 629-23 199)	19 823 (17 805)	10 138 (8758-11 517)	10 138 (1951)
Rivaroxaban-hemorrhage	39 (39)	17 237 (11 944-28 006)	21 374 (14 323)	16 671 (13 487-21 723)	20 951 (14 457)
SMX/TMP-rash	39 (39)	10 521 (6704-16 374)	12 483 (9322)	10 334 (10 082-25 132)	17 607 (14 759)
SMX/TMP–acute kidney injury	38 (38)	8427 (6722-13 563)	16 139 (32 189)	9507 (6383-12 343)	11 250 (7310)
Clopidogrel–gastrointestinal hemorrhage	37 (36)	13 794 (10 234-18 581)	40 291 (140 512)	13 820 (10 704-41 710)	26 207 (27 742)
Warfarin–cerebral hemorrhage	37 (37)	20 362 (15 568-57 984)	49 258 (59 981)	27 636 (22 147-44 285)	31 356 (11 528)
Apixaban-hemorrhage	33 (33)	17 617 (12 145-28 731)	25694 (18 632)	27735 (14241-48712)	31 807 (21 240)
Warfarin-hematoma	33 (32)	15 251 (7703-18 278)	17 790 (15 624)	15 906 (15 773-16 248)	24 360 (25 472)
Atorvastatin-rhabdomyolysis	32 (32)	22 125 (12 374-43 877)	34 391 (30 920)	46 341 (18 541-66 985)	51 372 (33 657)
Metoprolol-bradycardia	32 (32)	8004 (6212-12 419)	14 282 (23 786)	11 196 (8236-13 120)	11 577 (4093)
Lisinopril–acute kidney injury	31 (31)	11 482 (7215-19 053)	15 752 (12 632)	12 837 (10 699-24 844)	18 195 (12 328)
Dabigatran-hemorrhage	30 (30)	12 502 (8612-22 196)	21 206 (24 420)	NA^b^	NA^b^
SMX/TMP–blood creatinine level increase	30 (30)	7456 (6534-11 932)	9930 (5492)	12 870 (8806-19 745)	14 275 (7878)
Clopidogrel-anemia	29 (28)	12 207 (9547-21 362)	17 221 (12 794)	10 704 (9547-11 860)	10 704 (1635)
Lisinopril–blood creatinine level increase	29 (29)	10 000 (5727-13 070)	17 444 (26 842)	10 000 (6265-10 699)	9288 (2713)
Warfarin-hematuria	29 (29)	21 909 (8215-35 058)	28 178 (25 485)	28 483 (9440-39 344)	29 583 (20 971)
Insulin-hypoglycemia	28 (28)	10 719 (7606-13 625)	14 506 (16 864)	9182 (8087-11 424)	9681 (1969)
Enoxaparin-hemorrhage	27 (27)	29 535 (21 231-44 236)	33 470 (17 043)	NA^b^	NA^b^
Losartan-angioedema	27 (27)	9638 (7239-28 894)	17 592 (14 697)	9220 (7547-9789)	10 454 (6088)
Glipizide-hypoglycemia	26 (26)	16 548 (8395-38 414)	27 252 (26 917)	16 334 (10 067-34 216)	22 142 (19 178)
Lisinopril-hypotension	25 (25)	8691 (6355-12 837)	15 945 (19 122)	11 550 (8696-12 837)	18 963 (21 326)
Rivaroxaban-anemia	25 (25)	15 613 (11 702-21 723)	17 922 (9597)	13 088 (10 388-21 723)	18 074 (11 847)

Abbreviations: FY, fiscal year; HCTZ, hydrochlorothiazide; MedDRA PT, Medical Dictionary for Regulatory Activities Preferred Term; NA, not applicable; SMX/TMP, sulfamethoxazole-trimethoprim.

^a^ Costs adjusted to 2018 US dollars using the Consumer Price Index.

^b^ There was only 1 drug-MedDRA PT pair in fiscal year 2018.

## Discussion

This quality improvement study provides pragmatic estimates for the costs of drug-symptom pairs for severe outpatient ADRs that resulted in or contributed to hospitalizations in a national health care system. At the drug-symptom level, results highlight potential cost differences for the same ADR due to different medications. This information may be used to describe the cost of specific severe ADRs more accurately (eg, hospitalization for gastrointestinal hemorrhage with warfarin was more costly than with naproxen).

Although previous studies evaluated ADRs resulting in hospitalization, aggregate costs were generally presented (eg, total cost per year, average cost per patient).^2,3,4^ We did not report the total cost of ADRs because we used spontaneous reports and ADRs are underreported.^6^ We also could not directly compare our costs with those from other studies given differences in populations, medications, and cost sources. Regardless, the costs of severe ADRs leading to hospitalizations were high. Limitations of our study included the inability to determine the proportion of inpatient costs attributable to the ADR, preventability or causality, whether underreporting of ADRs biased cost estimates, and generalizability to non-VHA health care systems.

Our study ascertained hospital costs for drug-symptoms pairs, linking spontaneously reported, severe, outpatient ADRs with national, inpatient-level data. This information may be used by decision makers to estimate the cost avoidance of interventions to reduce ADRs (eg, use of a newly developed direct oral anticoagulants dashboard).

## Notes

Supplement.
eMethods.
