# Supplementary material for: Estimated Costs of Severe Adverse Drug Reactions Resulting in Hospitalization in the Veterans Health Administration
Source: JAMA Netw Open. 2022 Feb 10;5(2):e2147909. doi: 10.1001/jamanetworkopen.2021.47909 (PMC8832171; doi:10.1001/jamanetworkopen.2021.47909)
Supplement: Supplement. — eMethods. [file jamanetwopen-e2147909-s001.pdf]

Supplemental Online Content

Aspinall SL, Vu M, Moore V, et al. Estimated costs of severe adverse drug reactions resulting in hospitalization in the Veterans Health Administration. *JAMA Netw Open.* 2022;5(2):e2147909. doi:10.1001/jamanetworkopen.2021.47909

eMethods.

This supplemental material has been provided by the authors to give readers additional information about their work.

© 2022 Aspinall SL et al. *JAMA Network Open.*

eMethods

Framework and Data Sources

This was a retrospective cost-identification study from the VHA perspective. We used a modified cost evaluation framework to estimate the total costs of health care resources utilized to treat outpatient ADRs that resulted in, or contributed to, hospitalizations within VHA.[1] Because this project was conducted to help inform the safe use of medications within VHA, we followed the Squire 2.0 reporting guidelines for quality improvement studies.[2] Initially, an ADR is identified and documented by a health care professional who specifies the drug and descriptors of the adverse reaction in VHA's electronic health record. New events may subsequently be submitted as a report to VA ADERS. These reports contain details about the patient, drug(s), event date (estimated date the ADR occurred), ADR symptoms (i.e., descriptors of the reaction), and setting. ADR symptoms are coded using the Medical Dictionary for Regulatory Activities (MedDRA) terms.[3]

ADR-associated health care resource utilization and total costs were identified using the VA Health Economic Resource Center (HERC) Average Cost Datasets for VA Inpatient Care. The HERC average cost datasets provide estimates on inpatient care costs by distributing aggregate-level cost estimates to encounter-level utilization; cost functions are based on Diagnosis Related Group (DRG) weights used by the Centers for Medicare & Medicaid Services, hospital stay characteristics, patient demographics, and other clinical information. Additionally, we obtained ICD-9/10-CM diagnosis codes (admitting and principal) for the hospital stay from the Managerial Cost Accounting National Data Extract.

Inclusion/Exclusion Criteria for VA ADERS Reports

Outpatient-onset ADRs reported as severe in VA ADERS from FY2014 to FY2018 (i.e., 10/1/2013-9/30/2018) were included (Figure 1). We limited our analysis to severe ADRs because these events likely

© 2022 Aspinall SL et al. *JAMA Network Open.*

required hospitalization. Severe ADRs are defined per VHA Directive 1070 as, "ADRs which include any serious outcome, resulting in life- or organ-threatening situation or death, significant or permanent disability, requiring intervention to prevent permanent impairment or damage, or requiring/prolonging hospitalization."[4] Duplicate/invalid reports, or reports without complete data, were excluded. We also excluded ADRs reported >1 year after the adverse event occurred to improve the likelihood of finding the corresponding VHA hospitalization.

Linking Hospitalization and Costs to ADRs

Drugs and associated ADR symptoms from VA ADERS were linked to the most likely corresponding VHA hospitalization and cost in HERC based on patient identifiers and proximity to the date of the ADR (Figure 1). First, we selected the hospitalization that included the ADR event date. If none was found, we linked the ADR report with an event date falling within 14 days prior to, or after, a hospital stay because event dates in VA ADERS may be estimated by reporters. Reports without a corresponding VHA acute care hospitalization were excluded, as were reports with multiple cost records for the same hospitalization. We also excluded two reports with non-descriptive ADR symptoms (e.g., therapeutic response unexpected) and 42 (0.8%) VA ADERS reports with ≥7 symptoms per medication because each ADR symptom would be part of a drug-symptom pair, which is the unit of analysis, with the same medication and cost of hospitalization.

Validation

A 5% random sample of the included ADR reports were reviewed by three Clinical Pharmacy Specialists, each reviewing 1/3 of charts and meeting regularly to discuss questions, to verify the accuracy of VHA hospitalizations linked to ADRs. Validation was conducted by manual review of electronic health records. 92.1% of the ADR reports were associated with the correct hospitalization.

© 2022 Aspinall SL et al. *JAMA Network Open.*

Analysis

Characteristics of the ADR reports, and corresponding patient characteristics, were described. We grouped MedDRA terms at the Preferred Term (MedDRA PT) level, as used in the analyses of FAERS data by Hoffman et al.[5] The MedDRA PT is a unique, specific medical term that can be used to describe a sign or symptom. Each medication in the ADR report was paired with each MedDRA PT in the report because it was not feasible to identify the main symptom responsible for the hospitalization without chart review (e.g., hemorrhage vs. acute kidney injury). The primary analysis summarized the costs of ADRs by drug-MedDRA PT (i.e., symptom) pair, using mean, median, and measures of variance (standard deviation [SD] and interquartile range [IQR]). Costs were adjusted to 2018 U.S. dollars using the Consumer Price Index.[6] The drug-MedDRA PT was the unit of analysis because we were interested in costs of hospitalizations due to ADRs associated with a specific medication(s), and reporters may use different MedDRA PTs for the same ADRs (e.g., hemorrhage, gastrointestinal hemorrhage, anemia). All analyses were performed using SAS version 9.4 (SAS Institute Inc., Cary, NC).

© 2022 Aspinall SL et al. *JAMA Network Open.*

References

1. Gyllensten H, Jönsson AK, Hakkarainen KM, Svensson S, Hägg S, Rehnberg C. Comparing methods for estimating direct costs of adverse drug events. *Value Health*. 2017;20(10):1299-1310.

2. Ogrinc G, Davies L, Goodman D, Batalden P, Davidoff F, Stevens D. Squire 2.0 (Standards for Quality Improvement Reporting Excellence): revised publication guidelines from a detailed consensus process. *Am J Crit Care*. 2015;24(6):466-473.

3. MedDRA. Medication Dictionary for Regulatory Activities. https://www.meddra.org/ (Accessed March 26, 2020).

4. VHA Publications. VHA Directive 1070. Adverse drug event reporting and monitoring. May 15, 2020. https://www.va.gov/vhapublications/publications.cfm?Pub=1 (Accessed March 26, 2021).

5. Hoffman KB, Dimbil M, Kyle RF, et al. A drug safety rating system based on postmarketing costs associated with adverse events and patient outcomes. *J Manag Care Spec Pharm*. 2015;21(12):1134-1143.

6. U.S. Bureau of Labor Statistics. CPI Inflation Calculator. https://www.bls.gov/data/inflation_calculator.htm (Accessed July 2, 2021).

© 2022 Aspinall SL et al. *JAMA Network Open.*
